# Using Surrogate Parameters to Enhance Monitoring of Community Wastewater Management System Performance for Sustainable Operations

**DOI:** 10.3390/s24061857

**Published:** 2024-03-14

**Authors:** Zhining Shi, Christopher W. K. Chow, Jing Gao, Ke Xing, Jixue Liu, Jiuyong Li

**Affiliations:** Sustainable Infrastructure and Resource Management (SIRM), UniSA STEM, University of South Australia, Mawson Lakes, SA 5095, Australia; linda.shi@unisa.edu.au (Z.S.); jing.gao@unisa.edu.au (J.G.); ke.xing@unisa.edu.au (K.X.); jixue.liu@unisa.edu.au (J.L.); jiuyong.li@unisa.edu.au (J.L.)

**Keywords:** community wastewater management system (CWMS), online, wastewater, UV-Vis, surrogate parameters

## Abstract

Community wastewater management systems (CWMS) are small-scale wastewater treatment systems typically in regional and rural areas with less sophisticated treatment processes and often managed by local governments or communities. Research and industrial applications have demonstrated that online UV-Vis sensors have great potential for improving wastewater monitoring and treatment processes. Existing studies on the development of surrogate parameters with models from spectral data for wastewater were largely limited to lab-based. In contrast, industrial applications of these sensors have primarily targeted large wastewater treatment plants (WWTPs), leaving a gap in research for small-scale WWTPs. This paper demonstrates the suitability of using a field-based online UV-Vis sensor combined with advanced data analytics for CWMSs as an early warning for process upset to support sustainable operations. An industry case study is provided to demonstrate the development of surrogate monitoring parameters for total suspended solids (TSSs) and chemical oxygen demand (COD) using the UV-Vis spectral data from an online UV-Vis sensor. Absorbances at a wavelength of 625 nm (UV_625_) and absorbances at a wavelength of 265 nm (UV_265_) were identified as surrogate parameters to measure TSSs and COD, respectively. This study contributes to the improvement of WWTP performance with a continuous monitoring system by developing a process monitoring framework and optimization strategy.

## 1. Introduction

Sewerage service is considered an essential service that is provided to the communities. It is typically regulated by the Water Industry Act, which oversees water utilities through licensing, customer protection and retail pricing. Water utilities are required to operate under a certified Environmental Management System and to provide the best value-for-money services. In other words, they are continually seeking better ways to improve wastewater treatment plant (WWTP) operations. Sewerage may comprise a mixture of domestic wastewater (from household toilets, sinks, showers and washing machines), industrial effluent, occasional run-off of surface water and groundwater that has infiltrated into the sewers. Major WWTPs can typically handle megaliters per day of sewerage and serve a hundred thousand residents [1]. For major operations, a multi-stage treatment process is employed, including screening/grit removal, primary treatment (off-tank or primary sedimentation tanks or aerated lagoons), secondary treatment (lagoons, activated sludge reactors or secondary clarifiers), filtration (screen filters, media filters or membranes) and disinfection (UV or chlorine), and is often well-equipped with a comprehensive online monitoring system to maintain the desired level of service.

Unlike major wastewater treatment facilities managed by large water utilities, which have the resources to support more advanced monitoring devices for operation and control, community wastewater management systems (CWMS) often operate on much smaller scales and may have limited resources [2]. CWMS is a wastewater treatment system generally for rural/regional areas (with smaller populations) with less sophisticated treatment processes and managed by under-resourced local governments or communities. A CWMS is used to collect, treat and dispose of or reuse wastewater produced in the communities. It encompasses a wastewater collection and sewerage system that transports wastewater to a WWTP for treatment, storage, and disposal or reuse. CWMSs include different types of wastewater schemes such as the Septic Tank Effluent Drainage Scheme (STEDS), a full sewer or a combination of the two. The majority of the existing CMWSs are STEDS, which uses on-site septic tanks connected to the community sewer system with a centralized wastewater treatment facility. Discharge from the septic tanks typically flows to wastewater pump stations (WWPSs) by gravity and then is pumped through sewer mains to the wastewater treatment center. The quality of the sewerage can be unpredictable and unwanted substances could affect the performance of the treatment process, e.g., by slowing down or stopping the biological activity of a biological treatment process [3].

A significant challenge can arise in wastewater plant operations when unwanted substances, particularly caused by illegal dumps of trade waste or unexpected events, are introduced into the wastewater [4,5]. To support the CWMS, there is a need to implement wastewater quality monitoring programs using the best practices as guidelines. The CWMS industry is undergoing a shift towards placing a greater focus on real-time operational monitoring in the WWTPs of CWMSs. As most of the plants are in regional areas, conventional monitoring techniques involving sending grab samples for standard laboratory analysis often lead to delays in the implementation of corrective action and prolong the recovery time during unexpected process upsets. Conventional lab-based physical and chemical tests are commonly used to analyze the grab wastewater samples for monitoring the wastewater quality. However, these standard laboratory analyses, such as total suspended solids (TSSs) and chemical oxygen demand (COD), involve significant manual handling and prolonged processing times for results. Moreover, data from the standard laboratory analysis may not truly represent variations in wastewater quality due to their low temporal resolution. In contrast, online wastewater quality monitoring provides continuous measurements which allows for real-time assessment. This method can significantly aid in treatment process improvement by identifying contaminant sources and assessing the trends of wastewater quality changes [6]. System designers and operators are faced with the challenge of implementing suitable monitoring systems into the WWTPs of CWMS to enhance operational controls and improve treatment performance. In recent years, there has been a growing interest in the use of online instruments to monitor the various wastewater quality parameters of CWMS but primarily focusing on simple parameters such as pH and temperature [7]. Advanced online instruments for monitoring the wastewater of CWMS allow rapid assessment of treatment systems for real-time process control [8,9]. Online wastewater quality monitoring enables a quick response to wastewater events, optimization of treatment processes and the development of surrogate wastewater quality parameters. This contributes to Sustainable Development Goal 6 on water and sanitation (SDG 6) by providing real-time data on wastewater quality to enable efficient management and the control of wastewater treatment [10]. There are various online monitoring tools available for wastewater monitoring, ranging from simple monitoring devices that determine basic water quality parameters such as turbidity to more sophisticated instruments such as a UV-Vis spectrophotometer that provides detailed and insightful analytical information [11].

A UV-Vis spectrophotometer has been used as a technique for the online monitoring of wastewater parameters. Studies have shown that it can be used to quantify parameters, including biochemical oxygen demand (BOD), chemical oxygen demand (COD), nitrate, nitrite, and total suspended solids (TSSs) in wastewater [12,13], with the assistance of data analytical methods including multiple linear regression and partial least squares [14,15,16]. Research and industrial applications have demonstrated that an online UV-Vis spectrophotometer has great potential for improving wastewater monitoring and the treatment processes. Even though there are studies on the development of surrogate parameters with models from spectral data for wastewater, most of the studies were lab-based [15,16,17] or utilized spectral data from a bench-top UV-Vis spectrophotometer [12,18,19,20]. In addition, most of the industrial applications of the online UV-Vis spectrophotometer have focused on large WWTPs [14,21,22]. There were very limited studies on the development of surrogate parameters for small-scale WWTPs [23], such as the community wastewater management system of using online UV-Vis spectral data, particularly using advanced data analytics. Small-scale wastewater treatment systems are often used for small communities in regional or remote areas, having unique characteristics that distinguish them from larger municipal systems.

In this study, data analytical techniques, such as partial least square and principal component analysis, and linear regression were used to develop the surrogate parameter for estimating the concentrations of COD and TSS. The surrogates were developed using the online UV-Vis spectral data of influent wastewater from a WWTP of a CWMS. This study focused on the COD and TSSs as both parameters are commonly used to assess the quality of wastewater. Prior to the surrogate development, the optimal measurement frequency was analyzed with a value density calculation method for the online monitoring of wastewater quality using an online UV-Vis sensor. This study aimed at directly using absorbance values of single wavelengths as surrogate parameters to monitor wastewater quality and support treatment process optimization.

## 2. Materials and Methods

### 2.1. Case Study Site

**Figure 1:**
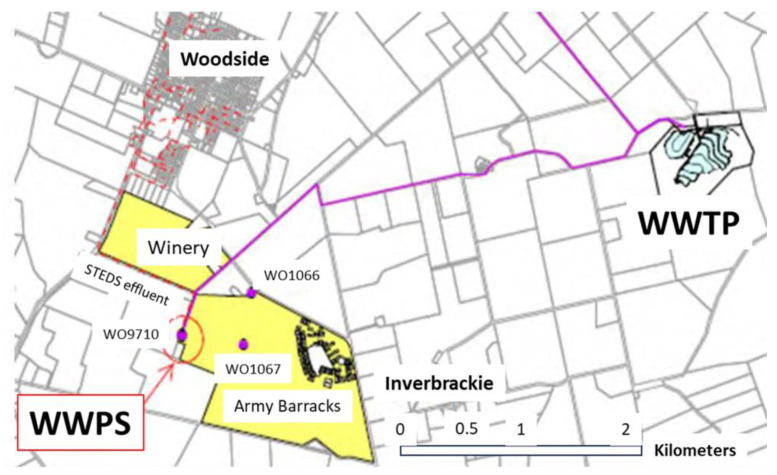
A map of the wastewater catchment, Wastewater Pump Station (WWPS) and Wastewater Treatment Plant (WWTP) locations.

The WWTP has a design capacity of 876 ML/year (current capacity of 420 ML/year), is situated in the Adelaide Hills, South Australia and it is close to the Woodside Township to serve approximately 5000 residents. The treatment processes of the WWTP include screens, grit removal, activated sludge biological nitrogen removal with chemical phosphorous reduction, secondary clarifiers followed by filtration and UV disinfection. Mechanical sludge dewatering is followed by anaerobic digestion on-site. The wastewater catchment is part of a large rural cesspool network with catchments, drawing from three main sources: Woodside, Charleston and Lobethal areas. The Lobethal Scheme is a traditional domestic sewerage catchment with long rising mains via a macerator and re-lift pump stations installed in the township of Lobethal to feed the WWTP as an independent supply. The second catchment, an integral part of a large council CWMS/STEDS catchment covers the Woodside and Charleston areas and primarily handles domestic wastewater. Moreover, the WWPS is located at the Woodside Army Camp on Wewak Road and is closely associated with the network of the second catchment, which enhances the overall efficiency of the wastewater management system. The catchment receives significant quantities of stormwater infiltration during seasonal wet weather events. A map of the wastewater catchment, WWPS and WWTP locations is shown in Figure 1.

The wastewater fed to the WWTP contains a high ratio of pathogens and nutrients, especially when receiving higher quantities of stormwater infiltration during seasonal weather events. This study site, Wastewater Pump Station, experiences inflows that are continuously variable in strength and chemical/biological characteristics. The discharge from the WWTP to Dawesley Creek was foul smelling and often green in color due to algal bloom in summer (December to February), causing ongoing concerns to the local Adelaide Hills Community.

### 2.2. Monitoring Location and Instrument

An online UV-Vis sensor, spectro::lyser™, from s::can Messtechnik GmbH, Austria was installed at the WWPS sewer well to provide the continuous monitoring of wastewater quality in influent intake for the WWTP. The WWPS sewer well is approximately 5.5 km from the WWTP. Designed for submersion, the s::can spectro::lyser™ is suitable for online and in situ monitoring applications. Its performance was enhanced by an automatic air cleaning system which used compressed air to periodically clean the sensor lens, ensuring accurate readings. This instrument operates on the principle of the photodiode array (PDA) spectrophotometer, notable for its lack of moving parts and the elimination of the need for chemical reagents. It features a durable and sensitive dual-beam UV-Vis sensor ranging from 200 nm to 750 nm, with optical path lengths in a selectable range of 0.05–10 cm. The path length selection is important to strike a balance between sensitivity and a wide response range. For this specific setup, a 2 mm path length was chosen to maximize measurement precision, demonstrating a tailored approach to achieving the best possible accuracy in monitoring.

The s::can spectro::lyser™ was configured to estimate the calculated equivalent of nitrate, total chemical oxygen demand (COD), soluble/filtered chemical oxygen demand (CODf) and total suspended solids (TSSs) using its proprietary algorithms. Local calibration (this is the terminology used by the instrument to describe the calibration and validation procedure) based on real media properties enables adjustment to align with laboratory results specific to each site. Calibration and validation procedures were conducted in accordance with the instrument instruction manual, as detailed in [24]. To ensure accuracy, a check sample was collected on 26 June 2014 (the start of this study) and sent to the laboratory for analysis using standard methods as part of the pre-work analytical quality control process. A weekly manual cleaning routine was implemented with milli-Q water to make sure the lens of the sensor was clean and minimize lens-surface fouling.

### 2.3. Data Collection and Pre-Treatment

The online UV-Vis sensor was programmed to monitor the wastewater quality of influent intake for the WWTP at two-minute intervals. An external modem, supporting a Global System for Mobile (GSM) modem, was connected to a central controller for remote operations facilitated through a Virtual Private Network (VPN), which also communicates with an external lift pump and associated PLC controller. This allows the system to regularly sample wastewater and deliver effluent samples directly to the sensor for measurement. The acquired data were saved to the hard drive of the instrument and allowed for manual downloads on demand, the generation of daily reports at prescheduled times or the provision of “near real-time” information to the operators. The full spectral data of the influent were saved into fingerprint (FP) files and PAR files in CSV format. The FP files contained the time series spectral data with absorbance values against wavelengths from 200 nm to 750 nm at 2.5 nm intervals. PAR files contain calculated equivalents of wastewater parameters based on the built-in algorithms of the instrument, including TSSs and COD. These files in CSV format of 6-month data from June 2014 to February 2015 (153,986 measurements/spectra) were imported into a database. All the FP files were merged into one FP file and all the PAR files were merged into one PAR file using R code developed in the previous study [11]. An error correction procedure was then applied to identify and rectify problematic data, including negative values, null values and extremely large values. These data are caused by the inaccuracies in measurements of the sensor and the sensor marks the status of these data as ‘errors’. The accuracies are caused by instrument failure due to electricity outages and a component of the instrument breaking down. Common data correction methods are applied to transform the data to make it more manageable and meaningful for analysis [25]. Negative values and null values were rectified by substituting them with the mean value of hourly data. Extremely large values were found as being several folds larger than other high values within the dataset [26] Extremely large positive values were replaced by the three-times standard variance of the hourly data (151,500 measurements were used for the study).

### 2.4. Data Analysis

The data pre-treatment and processing of FP and PAR data of the influent were conducted in the workspace of R and R-Studio. This case study covered 6 months and reprocessing the online data from June 2014 to February 2015 to capture the water quality changes for wet and dry seasons. The online UV-Vis spectra of influent wastewater quality data of the WWTP were utilized to visualize the trends of wastewater parameters, to build correlations between the parameters, to determine the optimal measurement frequency and to develop the surrogate parameters. The instrument measurement frequency optimization was conducted to find the ideal monitoring interval between two measurements and develop a measurement frequency recommendation. A value density calculation method was developed in Python to determine the optimal measurement frequency while maintaining the same degree of information; the actual measurement frequency of 2 min was compared against 10 min, 60 min, 120 min and 480 min using the full 6-month dataset (151,500 measurements/spectra data from 200 to 740 nm with 2.5 nm intervals). The maximum and minimum absorbance values were determined and divided into 200 bins in equal intervals to construct the Value Density Distribution (VDD) histogram; VDD of the two measurement frequencies (2 min vs. one of the lower measurement frequencies) were visually compared and the Value Density Distribution Error (SVDDE) was determined. The minimum measurement frequency with less than a 5% threshold of SVDDE was chosen as the recommended measurement frequency.

Partial least square regression (PLSR) was used to develop surrogate parameters for wastewater quality monitoring by determining the strongest correlation between single wavelengths and wastewater quality parameters including COD and TSS.

PLSR is an advanced technique that combines features from principal component analysis and multiple regression. It constructs components (latent variables) by projecting the observation and predictor variables to a new space. Then, linear regression models are built between new predictors and responses. Different from PLSR, principal component regression (PCR) builds linear regression models by constructing components without considering the response variable. PLSR and PCR are both suitable to model a response variable with a large number of predictor variables, where those predictors are highly correlated or even collinear. PLSR and PCR have been used for wastewater modelling in various studies, including the prediction of wastewater concentration and the improvement of the performance of wastewater treatment processes [27,28,29]. Online UV-Vis spectral data within wavelengths of 200–740 nm of influent wastewater and their corresponding wastewater parameters, TSSs and COD, were used to build PCR and PLSR models. The whole set of data was divided into train and test datasets using a randomization method with a ratio of 50:50 to develop and validate the developed models. The datasets were scaled to ‘0 to 1’ before they were used to develop PLSR and PCR models in Python.

## 3. Results and Discussion

### 3.1. Overview of the Influent Water Quality of the Wastewater Treatment Plant

**Figure 2:**
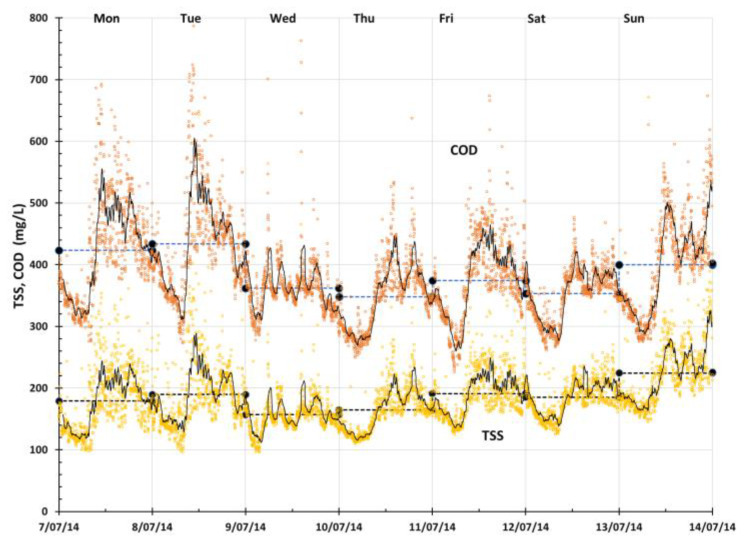
TSS and COD concentrations (2 min measurement frequency) from the online UV-Vis sensor with their hourly averages (black solid line) as well as their daily averages (black dots) over the 7 days. The daily averages can be viewed as a simulated equivalent if daily grab sample measurements of TSSs and COD were conducted using the standard laboratory monitoring procedure, as only one concentration was recorded to represent the concentration on that day.

An advanced online UV-Vis sensor was used to monitor the influent wastewater quality of the WWPS for 6 months from June 2014 to February 2015. Prior to this study, there was not much information available on the wastewater quality of the influent, which was a mixture of CWMS effluent and raw sewerage. This study was further developed after the previous work conducted by Adkins et al. [24] to provide a long-term evaluation of the suitability of the online UV-Vis sensor for influent wastewater quality monitoring. A previous study [30] showed that the influence of seasonal weather changes on the influent wastewater and dilution effects with ingress rainfall is significant and the discrepancy between online measurements and the laboratory was large. In addition, it was recommended by experts [31] that the site-specific calibration of the online UV-Vis sensor is conducted for measuring COD and TSS. To eliminate the calibration drift during wet weather conditions, the UV-Vis online sensor has been further calibrated with sufficient standard laboratory data of the grab samples of influent wastewater using the embedded calibration software (ana::pro, Version 5.0) from the instrument in this study. Figure 2 shows TSS and COD concentrations (2 min measurement frequency) from the online UV-Vis sensor with their hourly averages as well as their daily averages over the 7 days between 7 and 14 July 2014.

Figure 2 shows the data visualization of the daily and hourly averages of calculated equivalents for TSSs and COD generated from the online UV-Vis sensor as well as the laboratory data of TSSs and COD. It can be seen that the laboratory data of TSSs and COD are aligned well with the online data. Laboratory data of COD and TSSs fluctuated within the range of 330 to 450 mg/L and 150 to 230 mg/L, respectively, which also had low resolution due to the daily grab sampling and analysis, and was not able to represent the true variations in the wastewater quality. However, online data of COD and TSSs fluctuated within the range of 260 to 600 mg/L and 110 to 320 mg/L, respectively, and had high resolution. In addition, the online data of TSSs and COD, even after hourly averages, showed higher resolution and clear fluctuations of the wastewater quality within a week, which had much better representations of the wastewater quality trends. Studies have shown that site-specific calibration of online UV-Vis sensors enables accurate measurements of water quality by removing the interference of suspended particles on the UV absorbances [14,16,20,32,33,34].

Thus, the online UV-Vis sensor can give a continuous ‘near real-time’ fingerprint of the wastewater quality and enables showing the conditions of the wastewater quality through data visualization. Online monitoring using UV-Vis sensors can provide wastewater quality information in real-time and allow better process control of the wastewater treatment. This is particularly crucial for regional areas that experience increased vulnerability due to the more frequent occurrence of weather events and the unauthorized disposal of trade wastewater.

**Figure 3:**
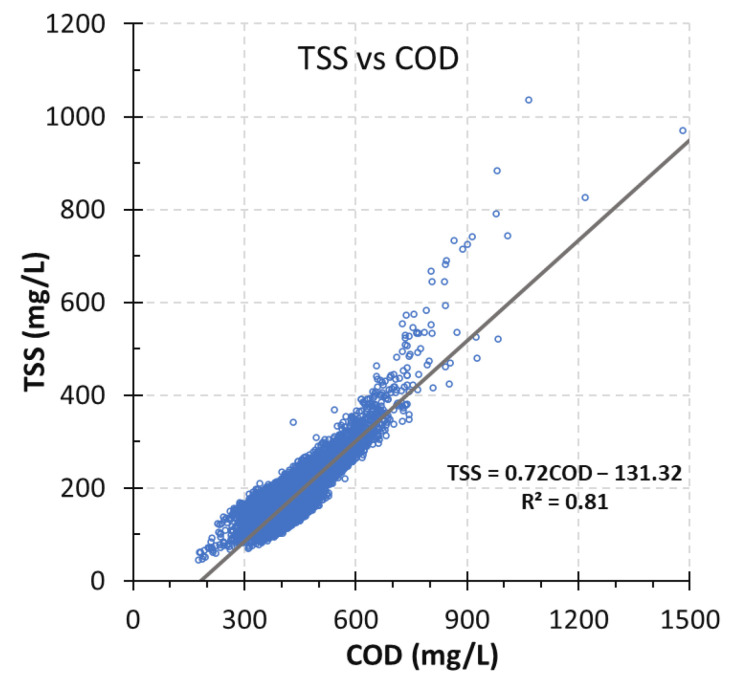
Relationship between COD and TSS concentrations in influent wastewater.

The relationship between COD and TSSs was explored using the data from the online UV-Vis sensor for the influent wastewater of the WWTP. A strong linear relationship between COD and TSSs was found with an R^2^ of 0.81, as shown in Figure 3. This finding was similar to other researchers’ work [35,36]. One study has found multiple regression among TSSs and COD, pH and total dissolved solids for the removal of solids from chemically enhanced primary treatment systems of wastewater [37]. Moreover, a linear correlation was found between COD and the total organic carbon for online monitoring of groundwater quality using an UV-Vis instrument [38]. This work provided valuable insights for assessing the wastewater treatment process. Thus, a conversion factor of 0.72 could be used to calculate the TSS concentration based on the concentration of COD for monitoring the TSS content in the influent wastewater. The relation between TSSs and COD in the influent wastewater was significant during the six-month study period. This indicates that the wastewater generally contains a high level of organics.

### 3.2. Measurement Frequency Optimization and Analysis

When operating an online UV-Vis sensor or other online instruments to monitor the wastewater quality, a critical consideration is to determine the optimal frequency to sample wastewater and capture a spectral reading, referred to as measurement frequency or sampling time (time between two measurements). If the measurement frequency is set too high, for instance, at one spectrum per minute, the resulting abundance of data records poses a substantial challenge in terms of data storage and analysis. It may become a significant burden to manage and interpret such a voluminous dataset. An extended interval between measuring points, such as a day, may raise concerns about overlooking vital water quality variations. There is no scientific consensus about a single method for measurement frequency optimization, but the minimum frequency that provides reliable estimates of the wastewater quality parameters is a goal of any water quality monitoring program [39]. It is crucial to find the optimal measurement frequency for capturing relevant data without overwhelming the online monitoring and process control system, ensuring comprehensive yet manageable monitoring with online UV-Vis sensors and similar instruments. A value density calculation method was developed to determine the optimal measurement frequency of the influent wastewater using an online UV-Vis sensor. The Value Density Distribution (VDD) of the two measurement frequencies were visually compared and absolute Value Density Distribution Error (SVDDE) was determined. Python was used to conduct the VDD and SVDDE calculations. Loops at three levels were built. The outer loops were on the measurement frequencies, the middle loops were on the absorbances at the wavelengths for the given rate, and the inner loops were for the given rate and absorbances at the given wavelengths.

The VDD of the sequence S for the n bins is shown below (details can be obtained from the Supplementary Materials):

VDD = [d_0,⋯d_(n − 1)]

**Figure 4:**
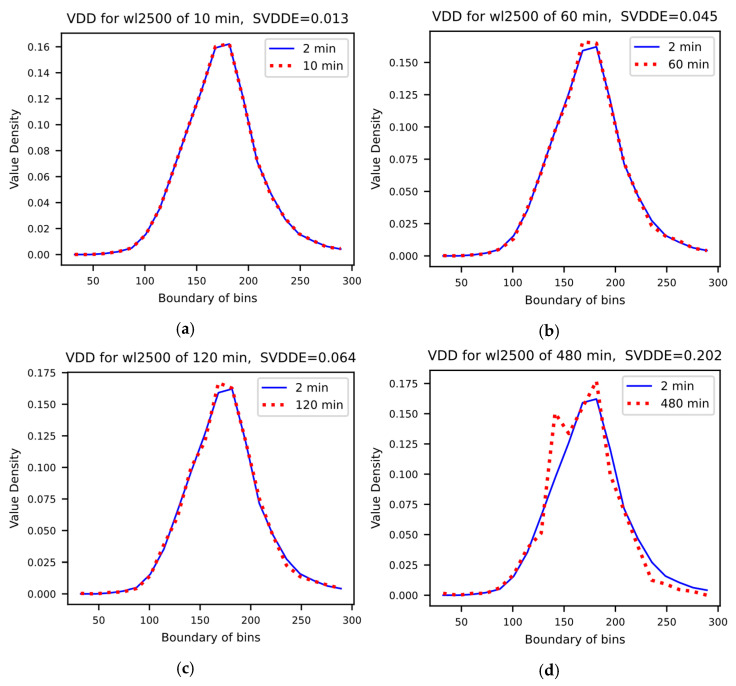
Value Density Distribution (VDD) plots with the calculated Sum of the Absolute Value Density Distribution Error (SVDDE) for absorbances at wavelength 250 nm (shown as WL2500 in the graphs) with a measurement interval of (**a**) 10 min, (**b**) 60 min, (**c**) 120 min and (**d**) 480 min, respectively.

The full spectral absorbance values of each UV-Vis spectrum (200–740 nm) are put into 200 bins with equal width. The sum VDD component is equal to one. Figure 4 shows the VDD for absorbance of the same wavelength, 250 nm, at various measurement intervals.

The first 20 bins of the VDDs with smaller values were plotted. The rest of the 180 bins with larger values had very small density values that were ignored in the plots. SVDDE value is the sum of the absolute differences between the blue solid line for two-minute samples and the red dotted line for g-minute measurements where g = 10, 60, 120 and 480 min. Figure 4a shows the comparison of VDDs of a two-minute measurement frequency (blue solid line) with that of a 10 min measurement frequency (red dotted line) for a wavelength of 250 nm. The trends of blue solid lines and the red dotted lines in the plots for measurement frequency of 10 min (Figure 4a) and measurement frequency of 60 min (Figure 4b) were quite consistent. With a frequency is 120 min (Figure 4c), the visual differences between the two VDDs became larger. With a frequency is 480 min (Figure 4d), the visual differences between the two VDDs were even larger. Therefore, it can be concluded that, for this case study, a measurement frequency of 60 min was the optimal instrument setting for monitoring the quality of influent wastewater.

**Figure 5:**
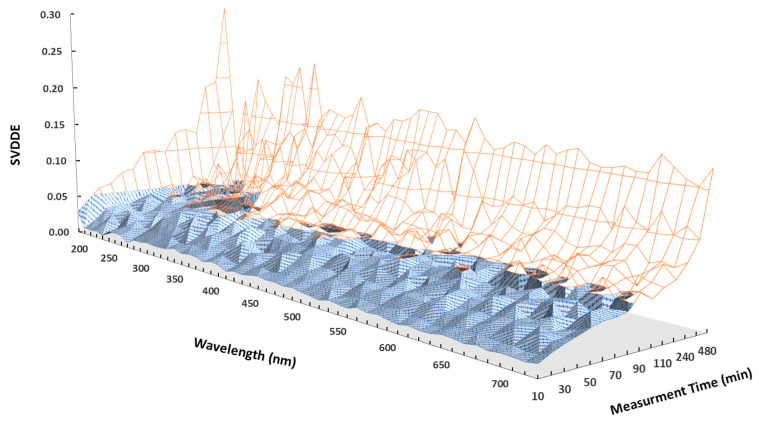
The Sum of the Absolute Value Density Distribution Error (SVDDE) of absorbances for wavelengths between 200 and 740 nm at various measurement times (10, 20, 30, 40, 50, 60, 70, 80, 90, 100, 110, 120, 240, 360 and 480) in minutes. The threshold is set at 0.05 (5% tolerance), and data in the blue color area are below the 0.05 threshold, which would be accepted as a suitable measurement frequency.

As the measurement frequency decreased from 10 min to 480 min, the errors across various bins (reflecting the distribution discrepancies) or the sum of SVDDE increased correspondingly, as stated in Figure 4 (10 min: SVDDE = 0.013, 60 min: SVDDE = 0.045, 120 min: SVDDE = 0.064 and 480 min: SVDDE = 0.202) and also shown in Figure 4. This is because when the measurement frequency became larger, the missed values became greater. A summary of the influence of measurement time on the SVDDEs for different wavelengths is shown in Figure 5. It can be seen that, generally, as measurement time increases, the SVDDE increases with some scattering for certain measurement frequencies.

Measurement times should be application-dependent. For example, if the 200 nm wavelength plays a very important role in an application, only a measurement time of 10 min per measurement collects a good representation of the two-minute measurements. But, for an application where the 280 nm wavelength is important, the measurement time can be 60 min per measurement. A tolerance threshold of 5% or 0.05 was set for the maximum acceptance of the SVDDE value, and the instrument measurement time of 60 min per measurement collected a good representation of the two-minute samples for most wavelengths. As data analysis was conducted using the wavelength ranges from 200 to 740 nm, the SVDDEs of all wavelengths within this range were all under 5%. It can be concluded that the optimal measurement frequency was 60 min in this study. This conclusion is supported by the research on the utilization of online UV-Vis sensors [11,25,40,41,42]. This research employed 60 min intervals of online UV-Vis measurements for water and wastewater quality monitoring as the hourly measurements and generated the most acceptable and reliable results. This procedure is recommended as part of the commissioning process to determine the measurement frequency to achieve the optimum instrument setup.

### 3.3. Development of Surrogate Parameters for Monitoring Wastewater Quality of CWMS

PLSR and PCR were used to develop the relationships between the absorbance of wavelengths and concentrations of wastewater quality parameters, TSSs and COD. PLSR and PCR have been used for wastewater modelling in various studies, including the prediction of wastewater concentration with conventional wastewater quality parameters, and monitoring and improving the performance of wastewater treatment processes [27,28,29,43]. These methods help to address the challenges of dimensionality and collinearity in wastewater quality analysis [44]. Wastewater data including UV-Vis spectra with calculated equivalents for TSSs and COD (proprietary algorithms after local calibration) were analyzed after the hourly average was applied. Hourly averages were used, as the previous section of this work concluded that the optimal measurement frequency was 60 min for monitoring the influent wastewater quality. TSSs and COD in the PAR files were generated from the online UV-Vis sensor after being calibrated with the standard laboratory data. The relationships between UV-Vis spectral data and wastewater quality parameters were explored. Single wavelengths had the strongest correlations with the parameters that were identified and selected as surrogate parameters for WWTP operations. Online UV-Vis sensors have built-in multivariate calibration algorithms based on PLSR for calibrations [21,22,45]. The optimal number of components (minimum number of latent variables) of the PLS and PCR models was found to be 10. It was established by leaving one out of cross-validation to avoid the under- or over- fitting of the model. Both PLSR and PCR achieved an R^2^ of 0.99 for wavelength selections using train and test datasets.

**Figure 6:**
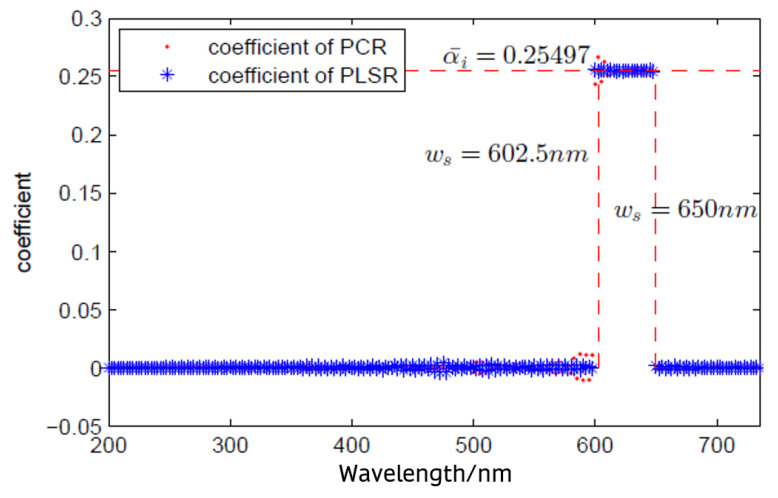
Analysis of contributions of absorbance of wavelengths to total suspended solid (TSSs) concentrations of influent wastewater using principal component regression (PCR) and partial least square regression (PLSR).

With both PLSR and PCR shown for the 217 regression coefficients of absorbances at wavelengths from 200 nm to 740 nm with 2.5 nm intervals and TSS concentrations of influent wastewater, only 20 of the regression coefficients were non-zero, and the rest of the coefficients approached zero for both PLSR and PCR. Furthermore, all the non-zero coefficients had the same values and were for consecutive wavelengths. Figure 6 shows an analysis of the contributions of the absorbance of wavelengths to TSS concentrations of influent wastewater using principal component regression (PCR) and partial least square regression (PLSR). Therefore, we conclude that the TSSs can be modelled in a linear function with the input of absorbances at 20 wavelengths between 602.5 nm and 650 nm.

The accuracies of PLSR and PCR models for TSSs and COD were evaluated, respectively, using the test datasets. The R^2^ of the prediction results show that PLSR and PCR models are good approximations for TSS and COD concentrations, ranging from 46.1 mg/L to 1364.7 mg/L and from 176.6 mg/L to 15,290.7 mg/L, in the influent.

**Figure 7:**
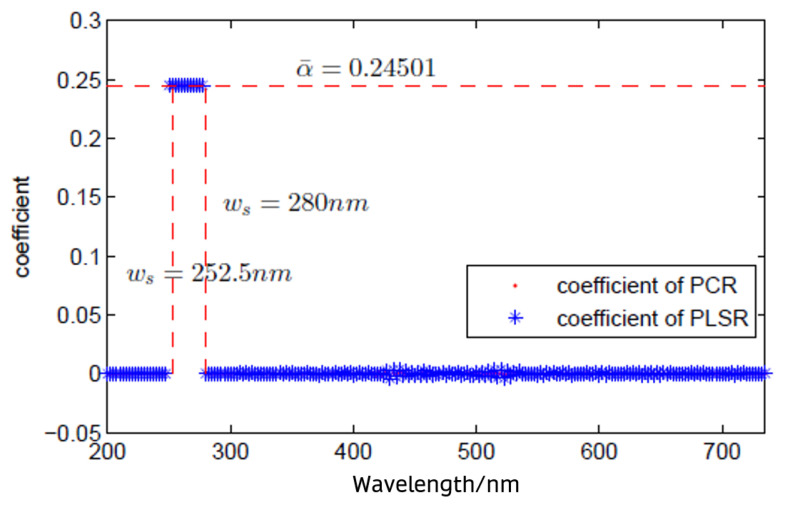
Analysis of contributions of absorbance of wavelengths contributing to chemical oxygen demand (COD) parameter concentrations of influent wastewater using principal component regression (PCR) and partial least square regression (PLSR).

The same procedure was applied to analyze the absorbance of wavelengths contribution to COD. The PLSR model and PCR model with 10 components were applied to fit the UV-Vis spectra and COD data of the influent wastewater. Similar to the regression models for TSS data, only 12 of the regression coefficients in the PLSR and PCR results were non-zero, which corresponds to a range of consecutive wavelengths with the same values. The regression coefficients of PLSR and PCR for COD are shown in Figure 7. It can be seen that the COD is modelled in a linear function with the input of absorbances at 12 wavelengths within the range of 252.5–280 nm.

**Figure 8:**
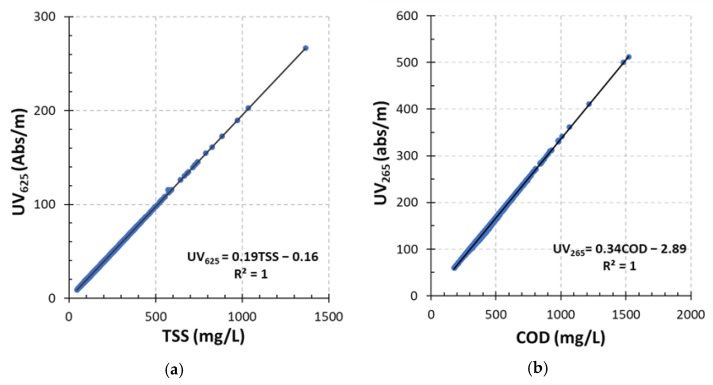
(**a**) Linear correlation between absorbance at wavelength 625 nm (UV_625_) and total suspended solids (TSSs) concentration of influent wastewater; and (**b**) linear correlation between absorbance at wavelength 265 nm (UV_265_) and chemical oxygen demand (COD) concentration of influent wastewater.

Further analysis with trial and error found that a perfect linear correlation was between absorbances at a wavelength of 625 nm (UV_625_) and TSS concentrations of influent wastewater with an R^2^ of 1, as shown in Figure 8a. Similarly, a perfect linear relationship was found between absorbances at a wavelength of 265 nm (UV_265_) and COD concentrations (Figure 8b). It can be interpreted as UV_625_ showing characteristics of TSSs while UV_265_ contains characteristics of COD. Thus, UV_625_ can be used as a surrogate parameter for measuring the concentration of TSSs in the wastewater and UV_265_ as a surrogate parameter for measuring the concentration of COD in the wastewater. Some researchers have been using surrogate parameters to predict the parameter changes instead of using standard laboratory analytical methods in the wastewater. Absorbances with shorter wavelength ranges have been used as surrogate parameters for rapid analysis and predicting contaminants in the wastewater for long-term monitoring [19]. Derivatives of a few wavelengths were utilized successfully to develop surrogate parameters for measuring the changes in natural organic matter [46]. In addition, absorbance at a wavelength of 190 nm was used as a surrogate parameter for measuring the COD concentration of wastewater [47]. Absorbance at a wavelength of 374 nm was used to estimate the COD concentration of treated wastewater [15]. The literature shows that an absorbance value of a single wavelength can be used as a surrogate to estimate the concentrations of wastewater quality parameters, but the selection of the wavelength depends on the wastewater matrix. The wastewater matrix can influence the wavelength selection for determining wastewater quality parameters due to its complex composition, which includes a wide variety of dissolved and particulate substances such as organic matter, inorganic ions and microorganisms. These components can absorb, scatter, or fluoresce light, affecting the measurement of specific water quality parameters.

**Figure 9:**
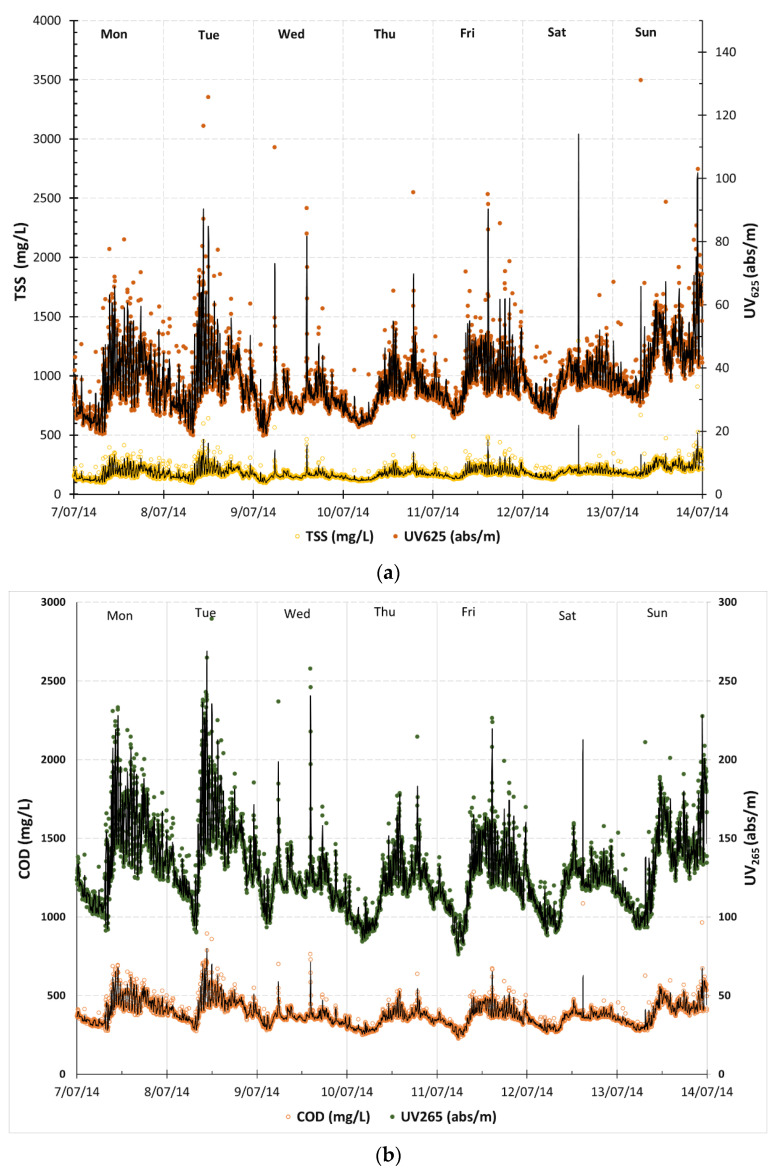
Comparison of trends in (**a**) absorbances at wavelength 625 nm (UV_625_) and total suspended solids (TSSs) concentrations of influent wastewater and (**b**) absorbances at wavelength 265 nm (UV_265_) and chemical oxygen demand (COD) concentrations of influent wastewater. Note: abs/m stands for absorbance per meter.

The trends of UV_625_ and TSSs in the influent wastewater were analyzed and are shown in Figure 9a using one-week data. The trend of UV_625_ aligns well with that of TSSs. The same approach was applied to UV_265_ and COD to compare their trends. It can also be inferred that the trend of UV_265_ closely correlates well with that of COD (Figure 9b). These findings further support the view that UV_625_ and UV_265_ can be used as surrogate parameters to measure TSSs and COD, respectively. The use of UV_625_ and UV_265_ offers rapid methods to monitor the variations in TSSs and COD in the wastewater, particularly for identifying deviations and detecting anomalies when unexpected shifts in raw water quality occur. Thus, simple UV-Vis sensors with fewer wavelengths could be employed in the field for water quality assessment, eliminating the need for more sophisticated full-spectrum UV-Vis instruments. Moreover, the use of single wavelengths as surrogate parameters may enhance the adoption of online UV-Vis sensors for continuous wastewater quality monitoring and process management by WWTP operators.

These online UV-Vis sensors can be located at raw wastewater intake and integrated with the supervisory control and data acquisition system. This integration facilitates the development of early warning and real-time process controls for wastewater quality management. By evaluating online surrogate measures, operators can promptly detect any aberrant changes, gauged by significant spectral variations, thus offering an early warning of rapidly changing wastewater quality. This capability enables operators to make immediate decisions regarding wastewater treatment processes in response to quality change events [25]. Overall, the online sensor can provide reliable surrogate measurements for continuous wastewater quality monitoring when the sensor is well-managed and calibrated correctly.

## 4. Conclusions

This case study focused on the WWTP in a regional community of South Australia to develop surrogate parameters for COD and TSSs by analyzing online UV-Vis spectral data of influent wastewater. Modelling techniques, including PLS, PCR and linear regression, were employed to establish the surrogate parameters. A key finding was the identification of the optimal 60 min measurement frequency for measuring influent wastewater determined using a value density calculation method. This measurement frequency was found to be the most effective for capturing the necessary data without overwhelming the system with too much information.

The study successfully identified absorbances at specific wavelength ranges using PLSR and PCR for developing surrogate parameters for COD and TSSs. For COD, wavelengths between 252.5 nm and 280 nm were deemed suitable. In contrast, for TSSs, wavelengths between 602.5 nm and 650 nm were identified. Notably, the research found perfect linear correlations: UV_625_ showed a direct correlation with TSS concentrations, and UV_265_ correlated with COD concentrations, both with an R^2^ of 1, indicating a flawless predictive relationship. The implications of these findings are significant. Thus, the ability to use UV_625_ and UV_265_ as surrogate parameters for measuring TSSs and COD, respectively, underscores the potential for increasing the utilization of online UV-Vis instruments for wastewater quality monitoring and process control. Such a development could simplify operations for WWTP operators, promoting more efficient and responsive management practices.

Furthermore, this study demonstrates the feasibility of developing an early warning system for community wastewater management systems using online UV-Vis sensors combined with advanced data analytics. It can also provide timely alerts for any unusual changes in wastewater quality and enables proactive responses to the treatment operation.

## Data Availability

The data that support the findings of this study are available on request.

## Supplementary Materials

The following supporting information can be downloaded at https://www.mdpi.com/article/10.3390/s24061857/s1; Figure S1: Hourly averages with daily trendlines for equivalent wastewater quality parameters, including TSSs and COD, generated from the online UV-Vis sensor; Figure S2: Value Density Distribution (VDD) plots with the calculated Sum of the Absolute Value Density Distribution Error (SVDDE) for absorbances at a wavelength of 265 nm (shown as WL2650 in the graphs) with a measurement interval of (a) 10 min, (b) 60 min, (c) 120 min and (d) 480 min, respectively; Figure S3: Value Density Distribution (VDD) plots with the calculated Sum of the Absolute Value Density Distribution Error (SVDDE) for absorbances at a wavelength of 625 nm (shown as WL6250 in the graphs) with a measurement interval of (a) 10 min, (b) 60 min, (c) 120 min and (d) 480 min, respectively.
