# Supplementary material for: Using Surrogate Parameters to Enhance Monitoring of Community Wastewater Management System Performance for Sustainable Operations
Source: Sensors (Basel). 2024 Mar 14;24(6):1857. doi: 10.3390/s24061857 (PMC10975892; doi:10.3390/s24061857)
Supplement: Supplementary file 1 [file sensors-24-01857-s001.zip › sensors-2883049-supplementary.pdf]

Supplementary Materials

Using Surrogate Parameters to Enhance Monitoring of Community Wastewater Management System Performance for Sustainable Operations

Zhining Shi, Christopher W.K. Chow*, Jing Gao, Ke Xing, Jixue Liu and Jiuyong Li

*Correspondence: christopher.chow@unisa.edu.au

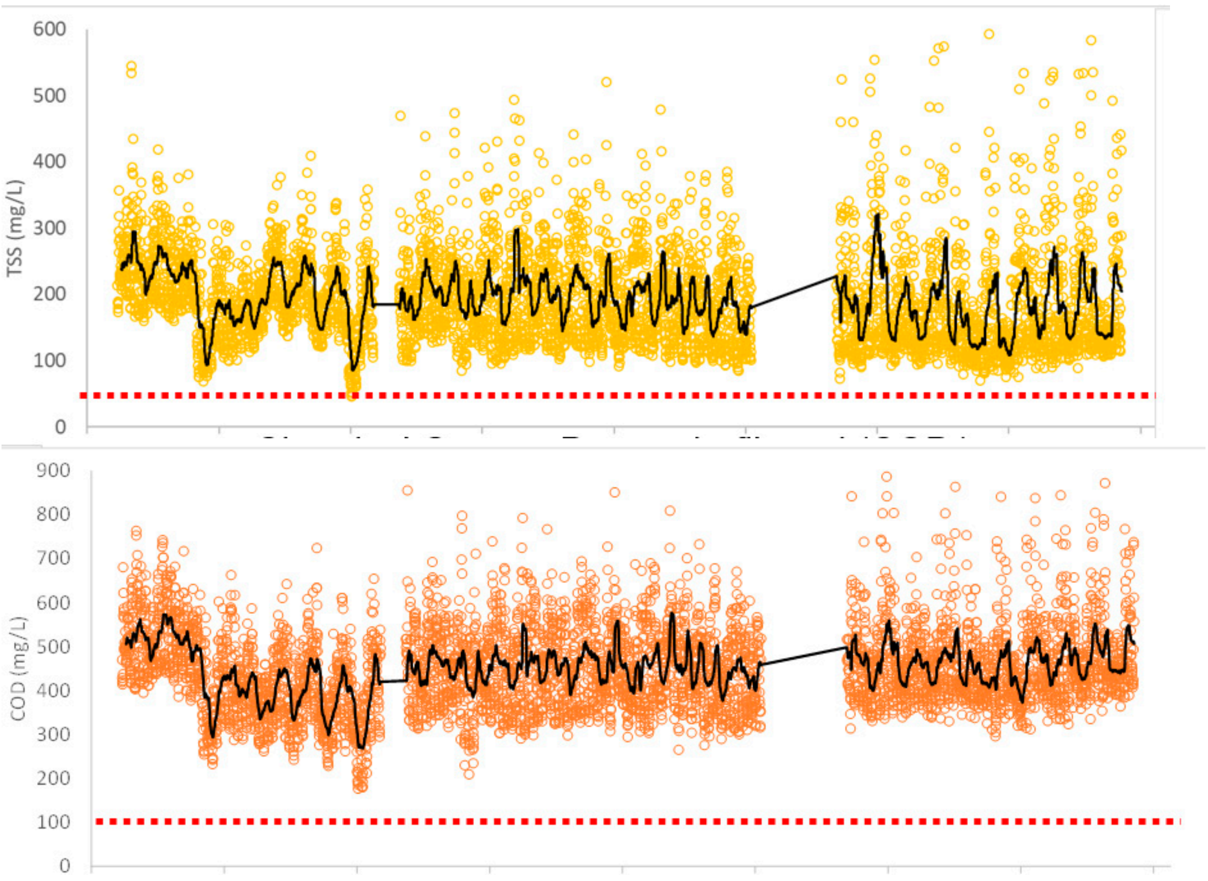

Figure S1. Hourly averages with daily trendlines for equivalent wastewater quality parameters, including TSSs and COD, generated from the online UV-Vis sensor.

Value Density Calculation and Terminology Definition

Given a value interval [v1, v2] called a bin, and a sequence S of sample values, the **value count** of the bin is the number of values falling into the bin in S. The **value density** is the value count divided by the total number of values in S. Two bins are neighbouring if their intervals share a boundary. Given n neighbouring bins, the value densities of all these bins form a distribution, called a **value density distribution**. The (value, count) pairs of the absorbances at a wavelength for a period can be used to determine the value density; for example, (600, 500) means that the absorbance value 600 appears 500 times in that period. The count is then divided by the total number of samples. The quotient is a type of density. Thus, the (value, count) pairs become (value, density) pairs and this process is the density calculation. Density calculation is completed for data obtained by measuring every two minutes and for data measured in other time intervals, such as 10 minutes, 60 minutes, 120 minutes and 240 minutes. After applying the moderation process from count to density, the (value, density) pairs of data in different measurement rates are in the same coordinates. As the time series of a certain wavelength has many different absorbance values and, therefore, many value pairs, they can then be plotted in a figure for comparison.
Value density analysis aims to determine if the value density distributions of two sequences of samples on the same set of neighboring bins are the same. The two sequences S_1 and S_2 are sampled from the same target with different sampling times. If their value density distributions are the same, the sequence with fewer values can represent the sequence with more values. In our study, S_1 is a one-minute sample sequence and S_2 is a g-minute sample sequence. If the value density distributions of S_1 and S_2 are the same, S_2 is a good representation of S_1. S_2 is derived from picking the first value in every g value in S_1 in order.
To determine if two distributions are the same, the density difference between corresponding bins from the two distributions can then be calculated. If the sum of the absolute differences in all bin pairs is less than 5%, the two sequences are taken as the same. After formally defining these concepts. Consider a sequence S of samples for a given wavelength w at a fixed sampling time (e.g., 2 minutes per sample) from a s::can spectro::lyser. With n bins, the following can be defined:

The width of each bin is $u = \frac{max(V)-min(V)}{n}$,
the interval of the ith bin is $b_i = [i * u, i * u + u)$,
the number of values of S falling in b_i: $c_i = count(v \in S \text{ and } v \in b_i)$, and
the value density of the ith bin is $d_i = \frac{c_i}{len(S)}$.

The value density distributions (VDD) are for the same wavelength but different sampling intervals. All values are put into 200 bins with equal width.

Wavelengths 265nm and 625 nm with various sampling intervals: The first 20 bins with smaller values are plotted. The other 180 bins with larger values have very small density values that are ignored in the plots. The SVDDE value is the sum of the absolute differences between the blue line for two-minute samples and the red line for g-minute samples, where g=10, 60, 120 and 480.

Value Density Distribution (VDD) for absorbance at a wavelength of 265 nm, at various measurement intervals, is shown in Figure S2.

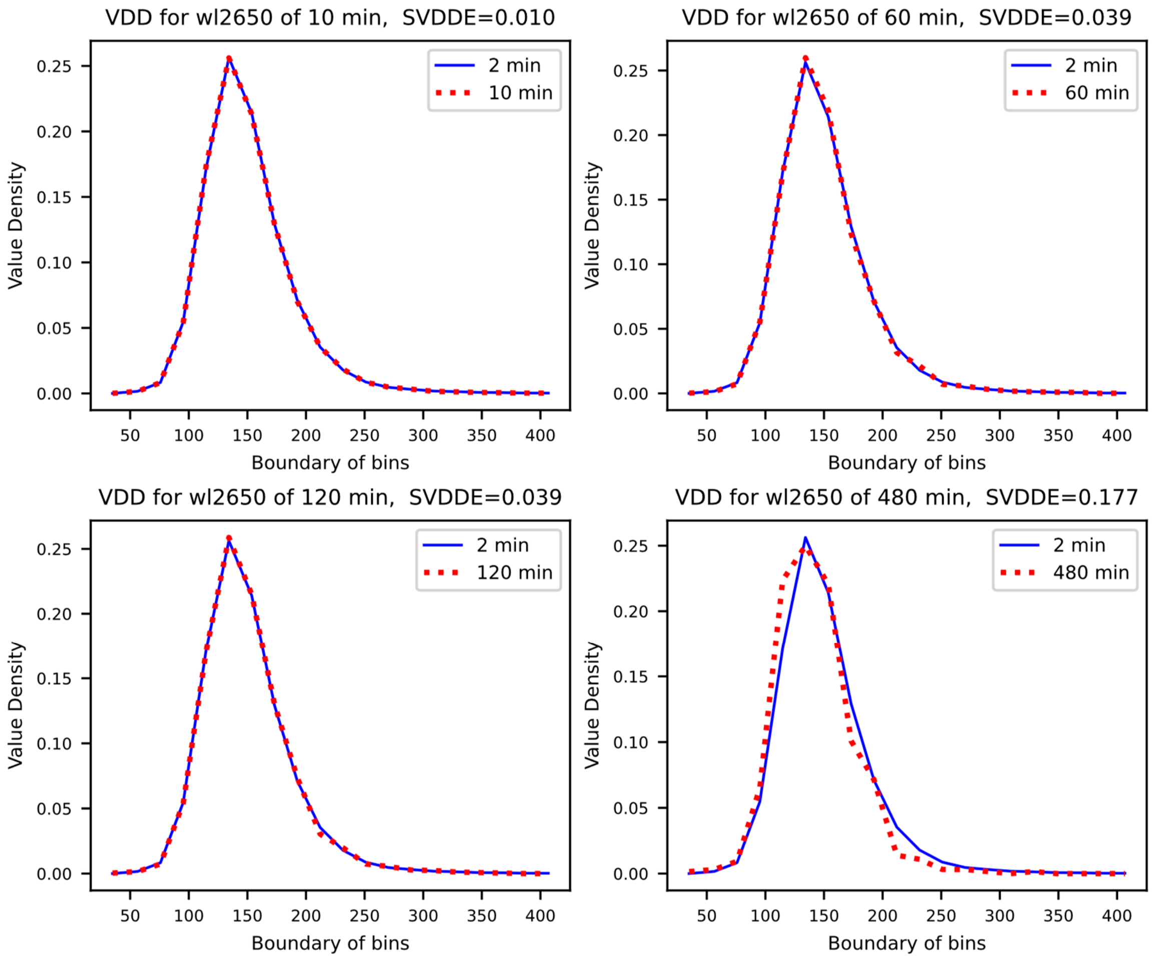

Figure S2. Value Density Distribution (VDD) plots with the calculated Sum of the Absolute Value Density Distribution Error (SVDDE) for absorbances at a wavelength of 265 nm (shown as WL2650 in the graphs) with a measurement interval of a) 10 minute, b) 60 minute, c) 120 minute and d) 480 minute, respectively.

Value Density Distribution (VDD) for absorbance at a wavelength of 625 nm, at various measurement intervals, is shown in Figure S3.

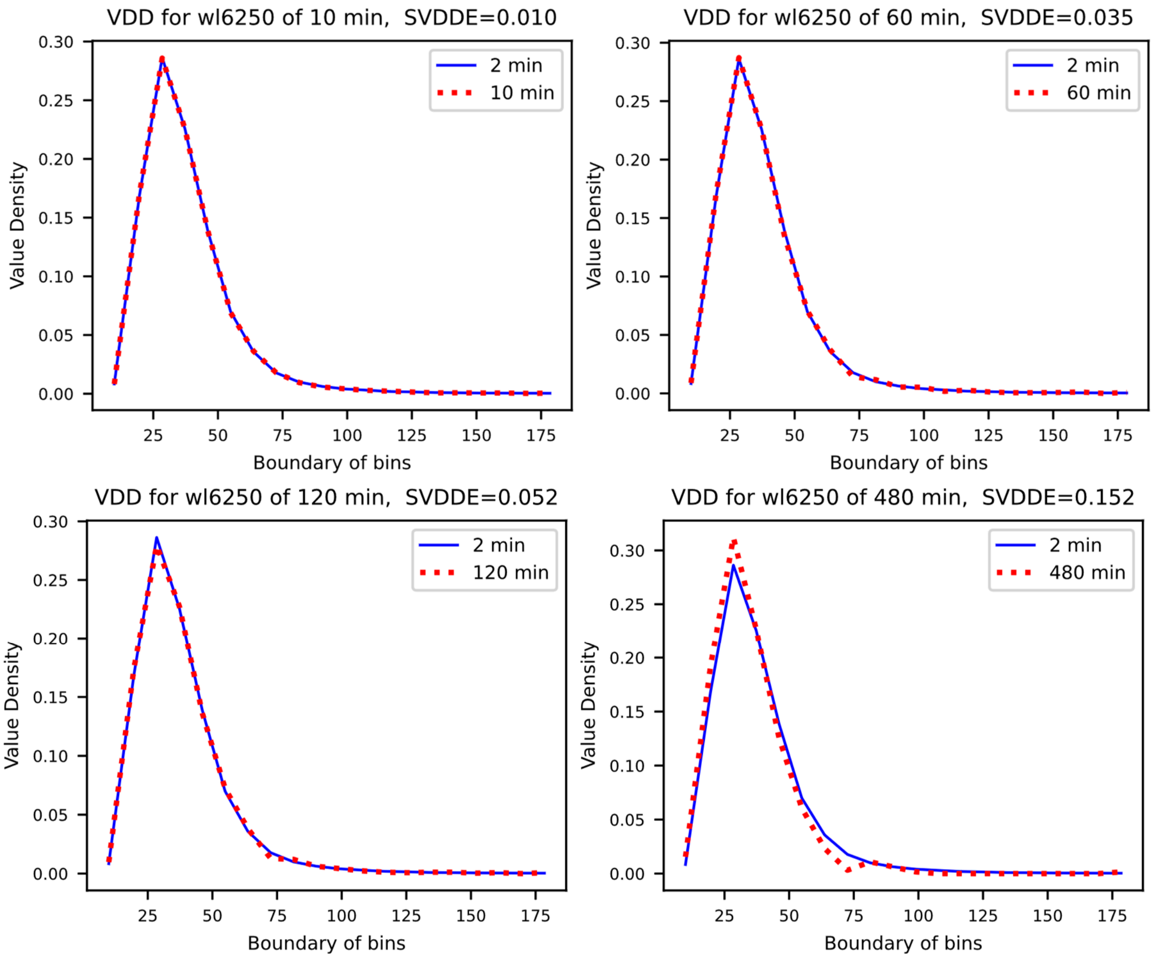

Figure S3. Value Density Distribution (VDD) plots with the calculated Sum of the Absolute Value Density Distribution Error (SVDDE) for absorbances at a wavelength of 625 nm (shown as WL6250 in the graphs) with a measurement interval of a) 10 minute, b) 60 minute, c) 120 minute and d) 480 minute, respectively.
